# Piloting short empathetic refutational interview modules in clinical training: Two UK studies^☆^

**DOI:** 10.1016/j.pecinn.2026.100455

**Published:** 2026-01-08

**Authors:** Dawn Holford, Emma C. Anderson, Harriet Fisher, Virginia C. Gould, Frederike Taubert, Linda C. Karlsson, Stephan Lewandowsky

**Affiliations:** a School of Psychological Science, University of Bristol, Bristol, United Kingdom; b Bristol Medical School, University of Bristol, Bristol, United Kingdom; c National Institute for Health Research Health Protection Research Unit (NIHR HPRU) in Evaluation and Behavioural Science (EBS), Bristol Medical School, University of Bristol, Bristol, United Kingdom; d Institute for Planetary Health Behaviour, University of Erfurt, Erfurt, Germany; e Department of Psychology and Speech-Language Pathology, University of Turku, Turku, Finland; f Department of Psychology, Åbo Akademi University, Turku, Finland; g Department of Psychology, University of Potsdam, Potsdam, Germany

**Keywords:** Vaccine communication, Healthcare professionals, Skills training, Empathetic refutational interview, Vaccine hesitancy

## Abstract

**Objective:**

Our objective was to introduce training in the Empathetic Refutational Interview (ERI), a novel framework for improving vaccine conversations and addressing vaccine misconceptions, as continuing medical education to improve HCPs' vaccine communication confidence.

**Methods:**

We introduced a short-form (60–90 min) ERI training module in two different UK clinical training settings: within a full-length immunisation training day (compared with a control communication module of the same length; Study 1) and as a stand-alone session (Study 2). We conducted a mixed-methods evaluation of training impact on participants' vaccine communication confidence.

**Results:**

Participants were HCPs who attended the training (Study 1: *n* = 61; Study 2: *n* = 98). Participants significantly improved their vaccine conversations after training. Control group participants described improved knowledge of information sources as supporting their confidence, while ERI group participants described improved communication skills and techniques. Participants reported that the ERI provided a conversation structure.

**Conclusion:**

Short training modules can improve HCPs' confidence in vaccine communication.

**Innovation:**

Our research applied an innovative new framework for vaccine communication training and produced novel insight on how this evidence-based communication structure helps HCPs gain awareness of effective vaccine communication skills, not just knowledge around signposting patients to information.

## Highlights

• Short training modules can improve HCP vaccine communication confidence.
• HCPs need training on how to communicate on top of knowledge of vaccine information.
• An evidence-based vaccine communication framework is helpful for HCPs.
• The Empathetic Refutational Interview can provide such a framework.

## 1 Introduction

Although vaccination is the safest and most effective way to protect against many diseases [1], vaccine hesitancy—the refusal or delay of available vaccinations [2]—threatens the success of global immunisation programmes. Declining vaccine coverage has led to outbreaks of vaccine-preventable diseases, e.g., measles outbreaks worldwide [3].

Healthcare professionals (HCPs) are trusted influencers whose recommendations can encourage vaccination uptake among hesitant patients [[4], [5], [6]]. Communicating effectively about vaccination can require HCPs to address misconceptions and false beliefs [7,8] while practising an empathetic and participatory engagement style [9,10]. However, it can be challenging for HCPs to communicate in this style [11]. HCPs often receive limited training in vaccine communication, with most educational interventions assuming that hesitancy is due to a patient's information deficits [10]. This gap in communication skills can affect HCPs' confidence and preparedness to recommend vaccination [12]. HCPs may even avoid difficult vaccine conversations if they suspect it might lead to conflict with their patient [11,13]. A strong recommendation from one's HCP is consistently found to increase vaccine uptake [14], so it is important that HCPs are confident and willing to recommend vaccination. Several studies have found that building HCPs' skills and confidence to deploy evidence-based best practice in communicating with vaccine-hesitant patients can lead to increased vaccination uptake (e.g., [6,[15], [16], [17], [18]]).

### 1.1 The empathetic refutational interview

One communication intervention that showed promising results in addressing vaccine hesitancy is Motivational Interviewing (MI): MI-trained HCPs have successfully improved the vaccine readiness of patients they speak with [17,18]. However, HCPs have reported wanting specific training to address vaccine misinformation [11,19], which MI does not address. Our study therefore used a communication framework designed to combine the strengths of MI with evidence-based psychological science strategies for refuting misconceptions: the Empathetic Refutational Interview (ERI) [20].

**Fig. 1:**
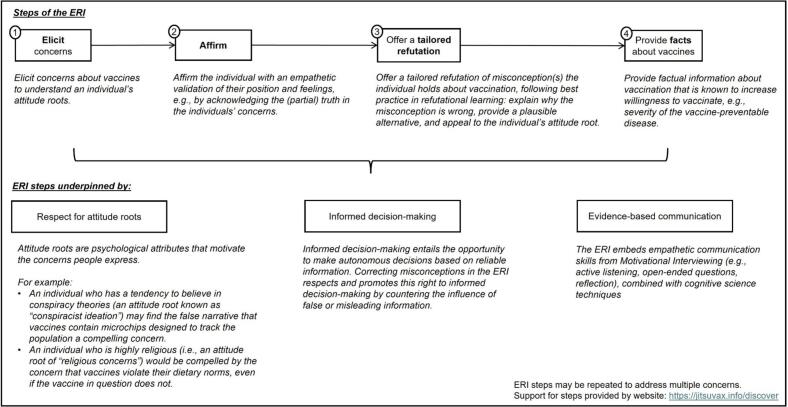
The four-step framework of the Empathetic Refutational Interview (ERI). *Note.* The description of the framework is adapted from [17].

The ERI is a four-step framework to guide conversations with vaccine-hesitant individuals (see Fig. 1). It incorporates principles of MI, taking a person-centred approach to elicit an individual's concerns and actively listen to, and affirm the individual. Beyond this, it provides guidance on psychological-science techniques for correcting misconceptions about vaccination (including misinformation). It can thus help HCPs with conversations where hesitancy is influenced by these misconceptions. One of the ERI's foundations is attitude root theory, i.e., that underlying psychological attributes (attitude roots) motivate specific concerns that individuals may express [21,22]. It is designed to help HCPs understand these different psychological underpinnings of vaccination (e.g., fears, distrust, and risk perception) and address these in their conversations with patients.

The ERI was found to gain more receptivity from vaccine-hesitant individuals compared to an information-giving approach [20] and HCPs also perceived the ERI as a better way to handle conversations with hesitant patients [23]. ERI-trained HCPs have increased patients' willingness to vaccinate and vaccine appointments booked after consultations compared to untrained HCPs [16]. However, it is not yet known how best to train HCPs to use the ERI approach.

### 1.2 Objective and overview of research

Our objective was to understand the impact of a brief ERI training module on HCPs' vaccine communication confidence and preparedness to address vaccine misconceptions that patients might bring up. We also assessed HCPs' qualitative feedback on the training.

We conducted two mixed-methods studies. In Study 1, we delivered the ERI module at the end of an immunisation training day and compared it to an existing communication module of the same length (control group). In Study 2, we delivered the ERI module as a stand-alone session with no control group. We evaluated changes in trainees' self-reported confidence and skills in each study. We also explored what elements in the modules support HCPs' learning. Across studies, we collected qualitative data for process evaluation, including questionnaire feedback on training experience and structured observations of the ERI training.

## 2 Study 1

### 2.1 Materials and methods

#### 2.1.1 Study context

We embedded the study within an in-person child immunisation training day run by a large regional NHS Trust in the South West of England (>15,000 staff and > 100 clinical services). Crucially, this training day contained an existing communication module that could serve as a control condition. While other health and public health organisations were approached and expressed interest in participating, none of these had an existing training programme (we addressed this wider interest in Study 2). Completion of the full day training was a requirement for staff in the organisation to be signed off to deliver vaccinations. It was also offered to other HCPs in the South West England region, who could pay to attend. The full day training covered theoretical and practical aspects of childhood immunisation and concluded with a 60-min module on communicating with patients and caregivers. Trainees (Table 1) were randomised to receive this (control) communication module or the ERI module. Data collection took place before and after the communication module. The study involved four separate training days between March and November 2023. Primary quantitative analyses were pre-registered on the Open Science Framework prior to data collection (https://osf.io/7a3rq).

**Table 1:** Breakdown of participants in each study and their characteristics.

	Study 1			Study 2
	Control	ERI	Total	
Number attended training, of which:	30	31	61	114
Consented to study	30	31	61	98
Completed baseline measures*	30	31	61	70
Completed post-training measures*	26	29	55	77
Completed 1 follow up*	20	21	41	40
Completed 2 follow ups*	18	13	21	31
Professional role:				
Nurse	29	31	60	33
Midwife	0	0	0	6
Community health worker	0	0	0	7
Admin/support role	0	0	0	9
Other roles**	1	0	1	8
Not reported	0	0	0	51
Age (in yrs)				
Mean (SD)	36.63 (9.08)	39.00 (11.12)	37.84 (10.15)	44.08 (13.52)
Median	34	37	37	43
Range	24–57	24–66	24–66	22–68
Years of clinical experience (in yrs)				
Mean (SD)	11.08 (7.72)	13.69 (9.88)	12.41 (8.91)	16.32 (14.61)
Median	9.50	10	10	13
Range	1–25	1–38	1–38	0–42
Gender				
Female	28	31	59	57
Male	2	0	2	6
Not reported	–	–	–	51
Ethnicity				
White	29	29	58	38
Black, African, Caribbean or Black British	0	0	0	16
Other ethnic groups	0	2	2	9
Prefer not to say/not reported	1	0	1	51
Vaccination role involves***:				
Scheduling appointments	4	7	11	28
Prescribing vaccines	0	3	3	3
Administering vaccines	20	17	37	13
Answering questions about vaccines	0	1	1	9
Documenting vaccines	0	0	0	1
No vaccination role	6	3	9	9
Not reported	0	0	0	51
Proportion vaccinated against influenza (at least once in past 3 yrs)	90%	97%	93%	80%

*Note.* Demographic information available only for participants who completed those fields. *Participants are recorded here as having completed post-test and follow ups if they provided at least one complete measure in these questionnaires. Some participants did not complete baseline questionnaires but did post-test questionnaires. **Other roles were: paediatrician, training consultant, health advisor, ***Multiple roles possible for each participant.

#### 2.1.2 Design and procedure

##### 2.1.2.1 Recruitment

Participants were recruited from among the trainees signed up to the full day training (either as a requirement for their job role or as an identified training need). Trainees were emailed information about the study before the training day. Participants gave consent before completing a baseline questionnaire before proceeding to the training day.

Participants attended the full training day all together and were randomly assigned to either the control or the ERI group for the final module, which the groups received in different rooms.^1^ After this module, participants completed a post-training questionnaire.

Participants were sent follow-up online questionnaires via an emailed link at one month and three months post-training. Participants were offered the chance to win a £50 shopping voucher for completing each follow-up questionnaire. Participants received three email reminders to complete each follow-up questionnaire, sent at one-week intervals.

##### 2.1.2.2 Training modules

***Control module***. This was an existing 60-min communication module for the immunisation training day. It consisted of a scenario-based exercise where trainees worked in small groups to discuss a scenario that featured a patient (or carer) with concerns about a vaccination, consider what the patient/carer might ask in a conversation and come up with responses to these. They then had a whole group facilitated discussion of their responses.^2^ The immunisation training co-ordinator delivered this module.

***ERI module.*** The 60-min ERI module consisted of a short presentation on the ERI framework, a demonstration video, an introduction to a supporting web resource (https://jitsuvax.info/discover), and two scenario-based exercises where trainees played the role of a HCP or patient to practise using the ERI in conversation, followed by a facilitated discussion. The scenarios were designed to be as similar as possible to the control exercise scenarios, while reflecting the ERI framework and the focus on a conversation. Two members of the research team (EA, HF) delivered the module. Researchers who delivered training by necessity were aware that they were delivering a training module. However data were collected using an anonymous online system so that researchers could remain blinded to individual participants' condition when performing analyses.

##### 2.1.2.3 Outcome measures

Our primary outcome measures were vaccine communication confidence (three items) and perceived preparedness to refute vaccine misconceptions (six items). Both were measured at baseline, post-training, and one and three-month follow-ups. Both measures used a five-point response scale with higher values indicating greater confidence and preparedness. For each measure, we computed a mean score for each participant. Full wording and descriptive statistics for these measures are provided in Supplementary Material.

***Vaccine communication confidence.*** We used three items from the “proactive self-efficacy” section of the International Professionals Vaccine Confidence and Behaviours questionnaire (I-Pro-VC-Be^12^), which ask about HCPs' commitment and preparedness to discuss vaccines with hesitant patients.

***Refutation preparedness for vaccine misconceptions.*** We selected six commonly encountered anti-vaccination arguments [24]. Participants rated how prepared they felt to respond to each argument if a patient brought it up.

***Secondary outcome measures.*** Participants also reported the number of influenza vaccinations they received in the last three years,^3^ self-reported understanding of ERI techniques, and frequency of use of ERI techniques. We report results for the secondary outcomes in Supplementary Material.

### 2.2 Results

#### 2.2.1 Participants

All trainees who attended the training day consented to participate (*n* = 61; participant demographics in Table 1).

#### 2.2.2 Quantitative analyses

**Fig. 2:**
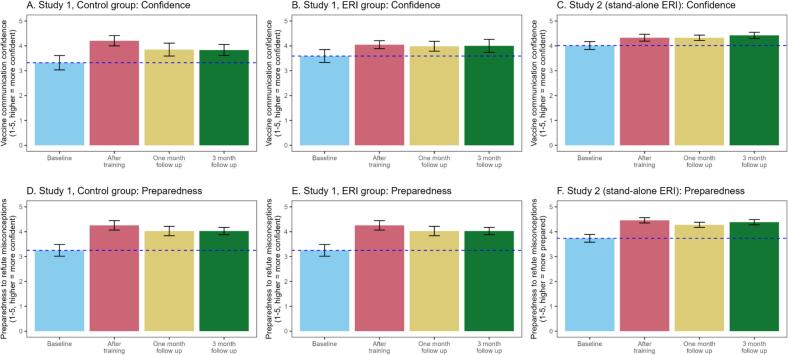
Confidence (top row) and preparedness (bottom row) at baseline and after training (immediately and at follow-ups) in Study 1 and 2. *Note.* Error bars indicate 95% confidence intervals. Dotted blue line indicates the mean score at baseline. (For interpretation of the references to colour in this figure legend, the reader is referred to the web version of this article.)

We ran pre-registered between-within analyses of variance (ANOVAs) on each primary outcome measure (Fig. 2 and Table 2), comparing study conditions (ERI vs. control; between-subjects) and timing (baseline vs. post-training; within-subjects). We then ran exploratory pairwise comparisons including the two follow-up time points.

**Table 2:** Means, standard deviations and 95% confidence intervals for confidence and preparedness at baseline and after training (immediately and at follow-ups) in Study 1 and 2.

	Baseline	After training	One month follow up	Three month follow up
Study 1				
Confidence				
Control	3.32 (0.80) [3.02, 3.62]	4.21 (0.57) [3.97, 4.44]	3.85 (0.72) [3.51, 4.19]	3.83 (0.62) [3.53, 4.14]
ERI	3.59 (0.73) [3.32, 3.86]	4.05 (0.46) [3.87, 4.23]	3.98 (0.56) [3.73, 4.24]	4.00 (0.75) [3.55, 4.45]
Preparedness				
Control	3.25 (0.66) [3.00, 3.50]	4.26 (0.53) [4.04, 4.47]	4.03 (0.53) [3.78, 4.28]	4.03 (0.41) [3.82, 4.23]
ERI	3.25 (0.71) [2.99, 3.51]	4.16 (0.39) [4.01, 4.31]	4.17 (0.47) [3.96, 4.39]	4.15 (0.61) [3.80, 4.51]
Study 2 (ERI only)				
Confidence	4.01 (0.87) [3.81, 4.22]	4.33 (0.75) [4.15, 4.51]	4.32 (0.59) [4.13, 4.52]	4.42 (0.68) [4.18, 4.66]
Preparedness	3.73 (0.85) [3.53, 3.94]	4.46 (0.59) [4.32, 4.60]	4.28 (0.56) [4.09, 4.46]	4.38 (0.59) [4.18, 4.59]

All participants' communication confidence improved significantly immediately post-training, *F*(1, 52) = 45.68, *p* < .001, η^2^_P_ = 0.47 (*d* = 0.90). This improvement was not significantly different between conditions, *F*(1, 52) = 2.84, *p* = .098, η^2^_P_ = 0.05 (interaction effect). For participants who completed the follow-ups, pairwise *t*-tests showed that confidence remained significantly higher than baseline at follow-up 1, *t*(40) = 4.22, *p* < .001, *d* = 0.66, and follow-up 2, *t*(30) = 2.88, *p* = .020, *d* = 0.52. Change in confidence did not interact with condition, *F*(3, 60) = 2.49, *p* = .070, η^2^_P_ = 0.11.

All participants' refutation preparedness increased significantly immediately post-training, *F*(1, 52) = 90.87, *p* < .001, η^2^_P_ = 0.64 (*d* = 1.31). This improvement was also not significantly different between conditions, *F*(1, 52) = 0.18, *p* = .676, η^2^_P_ < 0.01 (interaction effect). For participants who completed the follow-ups, pairwise *t*-tests showed that preparedness remained significantly higher than pre-test at follow-up 1, *t*(39) = 8.34, *p* < .001, *d* = 1.32, and follow-up 2, *t*(30) = 5.87, *p* < .001, *d* = 1.05. Change in refutation preparedness did not interact with condition, *F*(3, 60) = 1.68, *p* = .179, η^2^_P_ = 0.08.

## 3 Study 2

### 3.1 Materials and methods

#### 3.1.1 Study context

We organised ten ERI training sessions between June 2023 and May 2024 in London, the Midlands, and North West UK regions, each lasting 90 min. The module took approximately 60 min (same as in Study 1), with 15 min before and after for primary data collection. Health organisations and local authorities in each region advertised the sessions to HCPs in their catchment areas. Research team members (DH, HF) conducted the sessions in-person except for one delivered online at the request of the partnering health organisation.

#### 3.1.2 Design and procedure

All participants attended the ERI module, because no partner organisation could provide a comparative communication module—underscoring the training provision gap. Data collection procedures were similar to Study 1.

### 3.2 Results

#### 3.2.1 Participants

Ninety-eight trainees (out of 114) consented to inclusion in the study (see Table 1 for participant demographics).^4^

#### 3.2.2 Quantitative analyses

Analyses for this study focus on changes in the primary outcomes of vaccine communication confidence and preparedness to refute vaccine misconceptions (see Fig. 2). They were not pre-registered. We used paired samples *t*-tests to analyse baseline and post-training differences, followed by one-way within-subjects ANOVAs testing for changes over the follow-up period.

Compared to baseline, participants' communication confidence increased significantly post-training, *t*(57) = 2.80, *p* = .007, *d* = 0.37. The ANOVA across the follow-ups was not significant (*p* = .224).

Refutation preparedness increased significantly from baseline to post-training, *t*(57) = 7.42, *p* < .001, *d* = 0.97. The ANOVA across the follow-ups showed a significant overall change in preparedness, *F*(3, 36) = 13.46, *p* < .001, η^2^_P_ = 0.53. Pairwise comparisons showed that preparedness remained significantly higher at follow-up 1 and 2 compared to baseline, respectively: *t(*25) = 3.68, *p* = .004, *d* = 0.72; *t*(20) = 5.82, *p* < .001, *d* = 1.27.

## 4 Process evaluation

### 4.1 Materials and methods

Our process evaluation across both studies consisted of: feedback questionnaires collected post-training and at follow-up, and structured observations of the ERI modules. The evaluation question and observation schedule are provided in Supplementary Material.

#### 4.1.1 Feedback questionnaires

Participants were asked to give a quantitative rating of the informativeness, clarity, and usefulness of the session they attended immediately after training. This questionnaire invited open-ended feedback on: usefulness of training content, improvements in understanding, intended use in clinical practice, suggestions for workshop improvement and general feedback. At follow up 1 and 2, participants reported what training elements they had used in clinical practice and what they remembered from training. Data were analysed using a pragmatic qualitative content analysis [25]. A spreadsheet of free-text data was coded for response-types independently by two researchers (DH, EA), and final categories were confirmed via discussion. We used the tm and wordcloud packages in R version 4.3.1 [26,27] to generate word clouds as visual representations of the full set of free-text responses.

#### 4.1.2 Structured observations

A member of the research team completed an observation schedule for each ERI session to assess the fidelity of module delivery.

### 4.2 Results

We report here a content analysis of the free-text responses obtained across studies, highlighting differences between control and ERI participants. We then provide brief results of the structured observations. Broad categories of responses are reported in the text, with illustrative quotations presented in the respective tables (Table 3).

**Table 3:** Illustrative quotations reflecting themes from qualitative data on training experience.

Time & Group		Question category	Main category of responses	Illustrative quotation
Immediately after training	ERI	Useful aspects	Role play	It was useful to practice the techniques in a relaxed environment. It helped show how they can be used in practice just by changing your conversation approach slightly. *(Female, 14 yrs experience, nurse, ERI group)*
			(Four step) framework	I feel like this gave a good framework of how to use motivational interviewing type tools but in a very specifically vaccine-oriented way to discuss immunisation choices. *(Female, midwife, ERI group)*
			Specific ERI concept: Attitude roots	Belief system of the person having deep roots like a tree is a useful analogy. *(ERI group, demographic details not reported)*
			Specific ERI technique: Affirmations	I like the acknowledgement of the individuals concerns and the affirmation step. I thought this helps build trust with the individual which will help with their perceptions and views of healthcare. *(Female, 21 yrs experience, nurse, ERI group)*
			Specific ERI technique: Open questions	Open ended questions - I do use this approach but will try to listen more and potentially re- book appts to follow up. *(Female, 20 yrs experience, nurse, ERI group)*
			Specific ERI technique: Alternative narrative	I found the alternative narrative aspect of refutation very helpful to help move the patient towards the truth. *(Female, < 1 yr experience, administrator, ERI group)*
			Generally useful	All the elements taught was useful. *(Female, 14 yrs experience, nurse, ERI group)*
			Discussion	Discussions around how to approach different situations. *(Female, 34 yrs experience, nurse, ERI group)*
		Overall feedback	Generally positive	Really helpful to my role as a practice nurse and well delivered I will definitely use these new tools thank you. *(Female, midwife, ERI group)*
			Trainer expertise	Trainer is very knowledgeable and able to put across the process. *(Female, < 1 yr experience, administrator, ERI group)*
		Improvement	Longer session	Maybe a bit longer to go through more of the concerns and attitude roots. *(Female, 17 yrs experience, nurse, ERI group)*
			Earlier in the day	Timing being earlier in the day for brain capacity. *(Female, 7 yrs experience, nurse, ERI group)*
	Control group	Useful aspects	Discussion & peer learning	Everyone contributing and feeding back helped to broaden my understanding. *(Female, 11 yrs experience, nurse, control group)*
			Informational resources	Yes being able to signpost parent/patients to informations regarding vaccinations in children. *(Female, 8 yrs experience, nurse, control group)*
		Improvement	Longer session	Maybe a little longer and more scenarios. *(Female, 5 yrs experience, nurse, control group)*
Follow-up (one month & three months after training combined)	ERI	Used in practice	Applied ERI in clinical practice	Using the model to help organise the consultation, taking more of a step wise approach to discussions. *(Female, 21 yrs experience, nurse, ERI group)*
		Used in practice & remembered from training (combined)	Empathetic techniques	Using positive reinforcement and positive feedback, open ended questions and active listening to help facilitate positive conversations. *(Female, 6 yrs experience, nurse, ERI group)*
			Empathetic approach	Being more empathetic when having vaccine conversations. *(ERI group, demographic details not reported)*
			Attitude roots	I remember particularly the concept of attitude roots, looking at where an attitude has come from. *(Female, midwife, ERI group)*
			Passed on training	I've promoted the training and how it can help with conversations with the professional imms givers that I support. *(ERI group, demographic details not reported)*
		Specifically remembered from training	Role play	Role play was an excellent way to understand concerns and how to respond. *(Female, 31 yrs experience, nurse, ERI group)*
			Website resource	Tutorial on how to use the website. *(Male, 7 yrs experience, administrator, ERI group)*
	Control group	Used in practice	Informational resources	Before patients and families makes decisions about vaccines provide them with information on vaccines from a reliable source so they are able to give informed consent. *(Female, 8 yrs experience, nurse, control group)*
		Remember from training	Informational resources	That information is available for us to give to parents/patients that are unsure. *(Female, 21 yrs experience, nurse, control group)*
			Discussion & peer learning	The group discussion at the end was good to hear people's questions and their responses to real life scenarios. *(Female, 5 yrs experience, nurse, control group)*
	Both	Not used in practice	No opportunity/ exposure to vaccine hesitancy	I have not had the opportunity to discuss vaccines with patients. *(Female, 2 yrs experience, nurse, control)*

#### 4.2.1 Training feedback

**Fig. 3:**
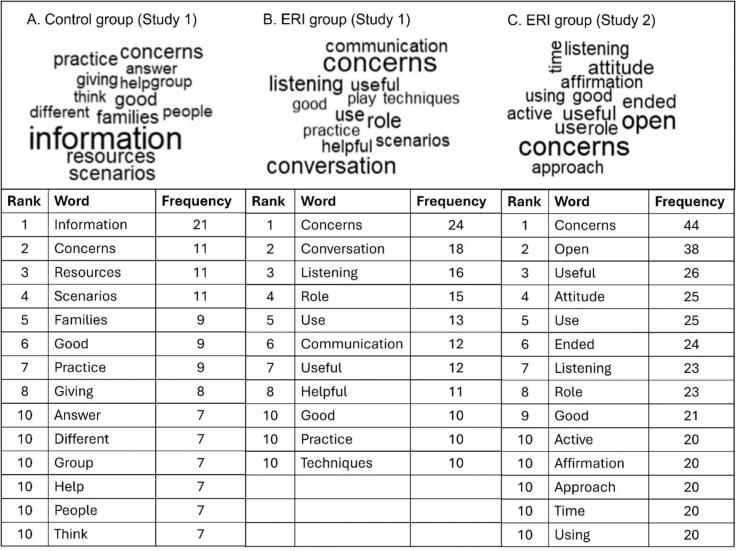
Word cloud and frequency tables showing the ten most frequent words in the open-ended responses from participants in the post-test and follow-up surveys. *Note.* For clarity of presentation, common English language words (e.g., prepositions), “vaccin*”, and overlapping words between groups (“patient”, “parent”, “session”, “questions”) are omitted. In the case of ties for ranked frequency, all words with the same frequency are included in the visualization.

Virtually all participants (130/131) ticked ‘yes’ to the question ‘did you find any elements of the workshop useful?’. Categories of free-text responses were similar across questions asking about usefulness of training content, improvements in understanding, intended use in practice and general feedback. As illustrated in Fig. 3, a key difference between groups was that control participants described information and resources they could give their patients, while ERI participants described skills and techniques they found useful for listening to patients and guiding conversations. Thus, the control group appeared to derive confidence from having vaccine information, while the ERI group from having a way to approach a vaccine conversation.

***ERI participants*** highlighted the role play exercise as being helpful for understanding and appreciated the interactive approach and group discussion. Participants valued the framework provided by the ERI and intended to use it in practice. Many highlighted specific ERI concepts/techniques, especially affirmations and attitude roots, with some mentioning open questions and the technique of providing an alternative narrative. There were many general comments stating the training was useful or mentioning improved understanding and confidence in how to approach vaccine conversations. A few participants mentioned the value of theory behind the ERI, and the website resource provided. Some intended to share the learning with others. General feedback was positive overall, with some appreciating the trainer's expertise. The main suggested improvements were to have a longer session and more scenarios. Many comments suggested no improvements were necessary. A few participants wanted more facts to counter specific myths/conspiracies, or extra resources (e.g., attitudes roots handout).

***Control group participants*** mainly valued the discussion and peer learning, especially any tips on communication from experienced trainees. They also valued informational resources. They expressed confidence from being able to answer patients' questions with facts or refer patients to information sources, which they intended to use in practice. General feedback was positive and suggestions for improvements were for a longer session and more scenarios.

Both groups commented that the role plays were helpful, though one ERI participant stated that they do not find role plays helpful, and one control participant reported being unclear on what was expected within the scenario work.

***Practical issues.*** Some comments across both groups alluded to specific practical issues on the day (e.g. timing mix-up, access issue). Some (study 1) participants mentioned the session would have been better earlier in the day, and some commented on the earlier training rather than the conversation module.

#### 4.2.2 Follow up (both time points combined)

***ERI participants*** commented that they had applied the ERI framework within their clinical practice. Many mentioned the specific techniques including active listening, open questions, affirmation and taking an empathetic approach to understand patient concerns; several mentioned attitude roots in this context. A few mentioned they had passed on the concepts to others. Similar comments were given when participants were asked what they remembered from training, with the addition that many mentioned the valuable role play, and some the website resource.

***Control participants*** commented that they had used information and resources in their clinical practice. When asked what they remembered from training, they similarly mentioned vaccine information and facts. Many mentioned the discussion, peer learning and the value of understanding different perspectives and responses. Two mentioned role play.

Where participants across both groups said they had not used the skills in clinical practice, the reason given was always because they had not been exposed to vaccine hesitant people in their role in the intervening time.

#### 4.2.3 Structured observations

Observers rated module fidelity as good-excellent: all trainers covered the planned content and provided sufficient time to complete and discuss the training exercises. Observers corroborated participant feedback about course duration and timing. They noted that trainees might need more time to grasp communication skills: trainees tended to jump quickly to correcting misconceptions and providing facts when role-playing, even when explicitly instructed to focus on eliciting concerns and affirming. Observers also reflected how the module timing within the training day sometimes constrained the time available when earlier sessions in the day ran late.

## 5 Discussion and conclusion

### 5.1 Discussion

Our objective was to understand if a brief ERI training module would have an impact on HCPs' vaccine communication confidence and preparedness to address vaccine misconceptions. Previous work suggests that HCPs' self-reported confidence is an important starting point for training since it affects how much they recommend vaccines [12]. We showed in two studies that HCPs reported higher confidence and preparedness for vaccine conversations after short sessions about the ERI (compared to before). These improvements were comparable between the ERI and control group. However, based on HCPs' comments, we identified two different ways that the training could improve confidence for HCPs: gaining knowledge so they can give patients information, and gaining awareness of communication tools they can use to improve conversations. This was a key difference between HCPs in the control group (who tended to report the former) and the ERI group (who tended to report the latter). Increasing HCPs' awareness of communication tools (not just vaccine knowledge) is particularly important in light of concerns that educational interventions for HCPs treat vaccine hesitancy as an information deficit problem that can be fixed through providing patients with information [10]. Other research has shown that having skills and techniques to guide conversations that maintain trust is linked to beneficial health outcomes for patients [28].

The additional value of the ERI module appeared to be in providing structure (through the framework) to guide conversations and a novel concept (attitude roots) that helped participants to understand patients' perspectives. These may not have been the only contributors to learning: there were other differences between the modules that could have contributed differently to confidence improvement (including the way the scenario-based exercise was executed: as a group exercise in the control; as paired conversation practice in the ERI). Nonetheless, ERI participants' qualitative comments indicated that the ERI framework provided additional scaffolding for their exercise. This was highlighted as valuable in enabling trainees to practise new skills within a controlled and supportive environment—which is an important aspect of medical education [29]. However, participants frequently suggested that training could be improved with more time to practise, which may indicate that the brevity of the module may not fully prepare trainees to put new skills into practice. This may also have been why we did not see greater changes in confidence relative to the control group: HCPs gained confidence from having new knowledge (evident in both groups), but not necessarily from securely acquiring the new skills. We thus posit that the main value of a brief training module is to raise HCPs' awareness and initial confidence to engage in empathetic and dialogue-based communication approaches, with further training likely necessary to strengthen HCPs' skills.

### 5.2 Limitations and recommendations for future research

One limitation in comparing self-reported confidence gains across groups in Study 1 is that participants also spent the training day learning about immunisation theory and practice. Given how participants reported knowledge as confidence-building, earlier learning in the day may mask the contributory effect of the communication-specific module. Study 1 was also conducted almost exclusively with nurses (reflecting the role profile of HCPs who require the immunisation training day for their role), which may make it harder to ascertain if we would find the same results for HCPs with different roles. It is promising then that Study 2's participants, who completed only the ERI module, and included HCPs in other roles (e.g., midwives, community health workers), also reported improved communication confidence. Confidence could be a function of learning what to communicate as well as how, with the ERI module showing promise for building HCPs' confidence and preparedness while guiding them towards more effective communication styles for vaccine hesitancy [30].

Another limitation was that although we measured improvements in confidence and preparedness, we were unable to evaluate if skills also improved, as we were unable to assess actual conversation skills due to time constraints. An open question is what minimum time commitment is required for effective skills acquisition. Longer interventions in continuing medical education are associated with stronger effects on trainees [31], with effective training to implement approaches such as MI often requiring several days [32]. Existing research in this space has demonstrated that training in MI and ERI do improve clinical encounters, with downstream impact on indicators such as improving vaccine uptake (e.g., [[16], [17], [18]]).

Finally, it is possible that participants' existing motivation to attend communication skills training could impact the confidence improvements we observed. This is unlikely to have much impact on Study 1 since the communication module was embedded within the wider training programme that was essential for most HCPs as part of their vaccination roles. Motivations might affect improvements for HCPs in Study 2, where the ERI training was conducted as a stand-alone module and advertised as communications training. Nonetheless, it is still important to design and evaluate training for HCPs who are motivated to attend it, since a lack of communication confidence can hold HCPs back from initiating vaccine conversations and giving recommendations even when they know they should be engaging in these [6].

### 5.3 Innovation

Our research is the first to introduce the ERI, a conversation framework for addressing vaccine misconceptions based on psychological science and misinformation research, as a novel intervention within medical education in the UK. It is also the first to adapt this framework into short teaching modules and evaluating its impact in different HCP education settings. Our findings provide novel insight into different contributors from communications training to HCPs' confidence in vaccine conversations.

### 5.4 Conclusion

Short training modules on vaccine communication can improve HCPs' confidence and preparedness to have conversations with vaccine-hesitant patients. HCPs may gain confidence from having more knowledge or from more awareness of communication tools. The ERI served as an evidence-based framework to structure training that was well-received, though longer training time is necessary to support conceptual understanding and practise skills.

## Ethics and open science statement

The work was received ethical approvals prior to data collection from the University of Bristol School of Psychological Science Ethics committee (reference: 12008) and the UK Health Research Authority (reference: 318853). Study protocols were registered and documented on the Open Science Framework (doi:10.17605/OSF.IO/7A3RQ). Materials, data, and the code to derive the reported analyses are shared on the OSF (https://osf.io/h8sv2).

## CRediT authorship contribution statement

**Dawn Holford:** Writing – original draft, Visualization, Validation, Supervision, Software, Resources, Project administration, Methodology, Investigation, Formal analysis, Data curation, Conceptualization. **Emma C. Anderson:** Writing – original draft, Validation, Resources, Methodology, Investigation, Funding acquisition, Formal analysis, Data curation, Conceptualization. **Harriet Fisher:** Writing – review & editing, Resources, Methodology, Investigation, Funding acquisition, Conceptualization. **Virginia C. Gould:** Writing – review & editing, Project administration. **Frederike Taubert:** Writing – review & editing, Methodology. **Linda C. Karlsson:** Writing – review & editing. **Stephan Lewandowsky:** Writing – review & editing, Supervision, Funding acquisition, Conceptualization.

## Funding sources

This project has received funding from the 10.13039/100010661Horizon 2020 Research and Innovation Programme grant 964728 (JITSUVAX).

LCK is supported by funding from the Turku Institute for Advanced Studies.

HF acknowledges support from the NIHR Health Protection Research Unit in Behavioural Science and Evaluation at 10.13039/501100000883University of Bristol. The Health Protection Research Unit (HPRU) in Behavioural Science and Evaluation at University of Bristol is part of the National Institute for Health Research (NIHR) and a partnership between University of Bristol and UK Health Security Agency (UKHSA), in collaboration with the MRC Biostatistics Unit at University of Cambridge and University of the West of England.

## Declaration of competing interest

EA and GG are executive directors of JITSUVAX Training, a not-for-profit company delivering training based on the JITSUVAX project. SL is a non-executive director.

The research was conducted entirely before the incorporation of this company.

All other authors declare no competing interests.

## Appendix A Supplementary data

Supplementary material Image 1

## Footnotes

1 On two occasions, a randomly selected participant had to be moved to a different group to meet minimum training numbers for the control group. Excluding these participants from the data did not change any results.

2 While this exercise was completed in groups, we observed that some participants perceived this to be a form of role play.

3 We collected data on HCPs' vaccination status for influenza as Study 1 pre-registered an extra hypothesis that the training might impact HCPs' own vaccination behaviour. However, no significant changes were observed (reported in Supplementary Material).

4 However, only 58 completed the quantitative outcome measures in full at both pre- and post-test.
