# Supplementary material for: Piloting short empathetic refutational interview modules in clinical training: Two UK studies
Source: PEC Innov. 2026 Jan 8;8:100455. doi: 10.1016/j.pecinn.2026.100455 (PMC12825064; doi:10.1016/j.pecinn.2026.100455)
Supplement: Supplementary file 1 — Supplementary material [file mmc1.pdf]

Supplementary Material

This file provides supplementary material and analyses for the paper *Piloting short Empathetic Refutational Interview modules in clinical training: Two UK studies*.

Primary outcome measures

Table S1. Full wording of all primary outcome measures

Outcome measure	Items	Response options
Vaccine communication confidence<br><br>*Instructions for each item:*<br>Please indicate your agreement with the following statement:	I am actively involved in ensuring that my patients are vaccinated.<br>I feel comfortable discussing vaccines with my patients who are highly hesitant about vaccination.<br>I feel sufficiently trained on how to bring up the question of vaccines with hesitant patients.	1: Strongly disagree<br>2: Somewhat disagree<br>3: Undecided<br>4: Somewhat agree<br>5: Strongly agree
Perceived preparedness to refute vaccine misconceptions<br><br>*Instructions for each item:*<br>Please imagine that you are interacting with an individual who gives that argument against having a vaccine.<br><br>Please indicate how prepared you feel to respond to the individual who said this.	Medical authorities are overreacting, with vaccines being recommended for every minor illness now.<br>People are being offered too many vaccines nowadays, and this will overload their immune systems.<br>Instead of vaccines, people should improve environmental factors like good hygiene, healthy lifestyles, and protective measures against the disease.<br>I worry about experiencing side effects from the vaccine.<br>Vaccines should not be administered to vulnerable people, such as pregnant women, young children or patients with allergies.<br>Negative experiences and testimonies of injuries by patients should be prioritised when deciding whether or not to accept vaccination.	1: Very unprepared<br>2: Rather unprepared<br>3: Undecided<br>4: Rather prepared<br>5: Very prepared

Table S2. Mean scores and reliability (Cronbach's alpha) of primary outcome measures

Outcome measure	Mean (SD)				Cronbach's alpha			
	Baseline	Post-training	Follow-up 1	Follow-up 2	Baseline	Post-training	Follow-up 1	Follow-up 2
Vaccine communication confidence (maximum score = 5)								
Study 1	3.46 (0.77)	4.12 (0.52)	3.91 (0.64)	3.90 (0.67)	0.63	0.80	0.69	0.83
Study 2	4.01 (0.87)	4.33 (0.75)	4.32 (0.59)	4.22 (0.68)	0.75	0.84	0.50	0.73
Perceived preparedness to refute vaccine misconceptions (maximum score = 5)								
Study 1	3.25 (0.68)	4.21 (0.46)	4.10 (0.50)	4.08 (0.50)	0.87	0.86	0.83	0.88
Study 2	3.73 (0.85)	4.46 (0.57)	4.28 (0.56)	4.38 (0.59)	0.89	0.86	0.85	0.90

Secondary outcome measures

Table S3. Full wording of all secondary outcome measures

Outcome measure	Items	Response options
Vaccination status	How many times have you been vaccinated against **influenza** during the last three years?	0, 1, 2, 3
Self-reported understanding of ERI techniques<br><br>*Instructions for each item:*<br>Please rate how well you understand the following communication skill/technique	Active listening<br>Eliciting concerns with open-ended questions<br>Understanding motivations for vaccine hesitancy<br>Giving affirmations<br>Tailoring refutations to vaccine misconceptions<br>Responding to immunisation concerns with facts	1: I have not heard of it<br>2: I have heard of it but do not understand it<br>3: I understand it but have not used it<br>4: I understand it and have used it but would like to become more comfortable with it<br>5: I understand it and am comfortable using it.
Frequency of use of ERI techniques (follow ups only)<br><br>*Instructions:*<br>In the past 3 months, how frequently have you used this skill/technique when you talk to individuals with immunisation concerns?*	Understanding motivations for vaccine hesitancy<br>Eliciting concerns with open-ended questions<br>Active listening<br>Giving affirmations<br>Tailoring refutations to vaccine misconceptions<br>Responding to immunisation concerns with facts	1: I did not talk to any individuals with immunisation concerns<br>2: Rarely/Never (0-25%)<br>3: Sometimes (26-50%)<br>4: Often (51-75%)<br>5: Most of the time (> 75%)

*Question only asked if participant indicated they have heard of the technique.

Table S4. Number of flu vaccinations received in past three years.

**Number of flu vaccines received**	**Study 1**		**Study 2**
	**Control**	**ERI**	
Baseline			
0	3	1	14
1	2	3	7
2	6	5	8
3	19	22	41
No response	-	-	-
Follow-up 1 (1 month post-training)			
0	3	1	9
1	1	0	6
2	3	6	11
3	13	14	14
No response	10	10	30
Follow-up 2 (3 months post-training)			
0	2	0	6
1	0	0	6
2	4	5	5
3	12	8	17
No response	12	18	36
Effects over time, baseline vs. follow-up 1			
Difference between conditions	$F(1, 39) = 1.31, p = .259$		-
Difference in no. of vaccines over time	$F(1, 39) = 0.80, p = .377$		$t(24) = 1.44, p = .161$
Interaction effect	$F(1, 39) = 0.22, p = .646$		-
Effects over time, baseline vs. follow-up 2			
Difference between conditions	$F(1, 29) = 0.23, p = .634$		-
Difference in no. of vaccines over time	$F(1, 29) = 0.17, p = .682$		$t(27) = 1.07, p = .293$
Interaction effect	$F(1, 29) = 0.17, p = .682$		

Table S5. Self-reported understanding of ERI techniques

Outcome measure	Study 1			Study 2
	Control	ERI	Comparison between groups	
Baseline				
Understanding attitude roots	4.08 (1.00)	3.82 (0.77)	$t(45) = -1.05$, $p = .301$	4.53 (0.70)
Eliciting concerns	4.04 (0.73)	4.11 (0.74)	$t(50) = 0.33$, $p = .742$	4.60 (0.65)
Active listening	4.40 (0.96)	4.39 (0.69)	$t(43) = -0.03$, $p = .975$	4.74 (0.50)
Affirmation	3.56 (1.33)	4.04 (0.79)	$t(38) = 1.56$, $p = .126$	4.61 (0.62)
Tailoring refutation	3.60 (1.47)	3.54 (0.58)	$t(31) = -0.21$, $p = .839$	4.31 (0.77)
Responding with facts	4.16 (0.94)	3.93 (0.77)	$t(46) = -0.97$, $p = .336$	4.55 (0.63)
Follow up 1 (1 month post-training)				
Understanding attitude roots	3.42 (1.26)	3.86 (0.57)	$t(25) = 1.38$, $p = .179$	4.43 (0.69)
Eliciting concerns	3.89 (0.88)	4.19 (0.51)	$t(28) = 1.29$, $p = .208$	4.49 (0.51)
Active listening	4.42 (0.61)	4.43 (0.60)	$t(38) = 0.04$, $p = .969$	4.65 (0.48)
Affirmation	3.79 (1.13)	3.81 (0.75)	$t(31) = 0.07$, $p = .948$	4.32 (0.71)
Tailoring refutation	2.95 (1.39)	3.43 (0.87)	$t(30) = 1.29$, $p = .206$	4.16 (0.69)
Responding with facts	3.89 (0.81)	3.76 (0.62)	$t(34) = 0.58$, $p = .568$	4.38 (0.68)
Follow up 2 (3 months post-training)				
Understanding attitude roots	3.72 (0.67)	3.77 (0.93)	$t(21) = 0.16$, $p = .878$	4.58 (0.56)
Eliciting concerns	4.06 (1.11)	4.15 (0.69)	$t(29) = 0.30$, $p = .764$	4.61 (0.56)
Active listening	4.61 (0.50)	4.31 (0.63)	$t(22) = -1.44$, $p = .165$	4.70 (0.47)
Affirmation	3.78 (0.88)	3.85 (0.69)	$t(29) = 0.24$, $p = .810$	4.61 (0.56)
Tailoring refutation	3.50 (0.92)	3.46 (1.13)	$t(23) = -0.10$, $p = .920$	4.33 (0.69)
Responding with facts	3.72 (0.83)	3.85 (0.69)	$t(28) = 0.45$, $p = .653$	4.67 (0.48)

Table S6. Frequency of use of ERI techniques

**Frequency of skill use**	**Study 1**				**Study 2**	
	**1 month post-training**		**3 months post-training**		**1 month post-training**	**3 months post-training**
	**Control**	**ERI**	**Control**	**ERI**		
Understanding attitude roots						
0-25%	1	1	2	1	2	1
26-50%	6	5	3	2	5	4
51-75%	0	4	2	2	16	15
76-100%	2	0	1	0	8	10
No patients*	2	6	5	3	2	2
No response	19	15	17	23	37	38
Elicit concerns						
0-25%	2	2	1	1	3	1
26-50%	1	4	1	4	10	7
51-75%	4	6	4	2	10	11
76-100%	2	0	2	0	11	11
No patients*	4	8	5	4	3	2
No response	17	11	17	20	33	38
Active listening						
0-25%	0	5	2	2	2	0
26-50%	3	2	5	3	7	5
51-75%	5	6	0	3	15	14
76-100%	3	1	5	0	11	12
No patients*	7	6	6	4	2	2
No response	12	11	12	19	33	37
Affirmation						
0-25%	1	1	2	2	2	0
26-50%	5	2	3	3	5	6
51-75%	2	4	1	1	15	10
76-100%	1	1	1	0	8	14
No patients*	3	5	4	3	2	2
No response	18	18	19	22	38	38
Tailored refutation						
0-25%	0	2	1	1	2	1
26-50%	3	3	3	3	9	5
51-75%	0	3	3	0	11	10
76-100%	2	0	0	0	6	11
No patients*	2	3	4	3	3	2
No response	23	20	19	24	39	41
Responding with facts						
0-25%	0	2	1	1	1	1
26-50%	6	5	1	5	7	5
51-75%	1	4	4	0	9	10
76-100%	2	0	0	0	13	15
No patients*	3	3	5	3	3	2
No response	18	17	19	22	37	37

Note. *No patients = "I did not talk to any individuals with immunisation concerns".

Evaluation questions

Table S7. Full wording of all evaluation questions.

Question type	Items	Response options
Evaluation ratings	The workshop was useful preparation to deal with individuals' concerns about immunisation.	1: Strongly disagree<br>2: Disagree<br>3: Agree<br>4: Strongly agree
	The workshop did not adequately address my worries about responding to individuals' vaccine concerns. [reverse-coded]	
	The content for the workshop was informative.	
	The communication approach discussed in the workshop had clear steps for me to follow.	
	I plan to use the communication approach from the workshop in my future clinical practice.	
	I did not find the workshop helpful to understand individuals' concerns. [reverse-coded]	
Open-ended evaluation questions	Did you find any elements of the workshop useful?<br>*If yes:* Please provide further information below.<br>*If no:* Please describe what we could do to make the workshop more useful.	Yes/No<br>Free text
	Did you feel you that you improved understanding as a result of the workshop?<br>*If yes:* Please provide further information below.<br>*If no:* Please could you describe what we could do to improve understanding during the workshop.	Yes/No<br>Free text
	Are there any elements of the workshop that you will use in your future clinical practice?<br>*If yes:* Please describe below.<br>*If no:* Please could you explain why?	Yes/No<br>Free text
	Are there any other ways that the workshop could be improved? If yes, please provide details below.	Free text
	Do you have any feedback for the workshop? Please write it in the box below.	Free text
Follow-up evaluation questions	What do you remember most from the session?	Free text
	Are there any elements of the workshop that you have used in your regular/clinical practice?<br>*If yes:* Please describe below.<br>*If no:* Please could you explain why not.	Yes/No<br>Free text

Observation schedule

<table>
<tr><td>Date:</td><td>Location of session:</td><td>Time started:</td><td>Time finished:</td></tr>
<tr><td colspan="4">Overview of training session logistics: (e.g. space session delivered, who delivered the session, how were participants seated, how many attendees).</td></tr>
<tr><td>Criteria</td><td>Extent met *</td><td colspan="2">Descriptive account (summarise observational evidence to support the extent to which the criteria has been met)</td></tr>
<tr><td colspan="4">* Score on Likert scale: 1=Criteria not covered; 2=Criteria poorly covered; 3=Average: 4; Criteria covered well; 5=Criteria extensively covered</td></tr>
<tr><td colspan="4">Evidence for learning outcome being met</td></tr>
<tr><td>Knowledge about motivating factors for vaccination concerns</td><td></td><td colspan="2">e.g. Did participants engage well in the exercise about attitude roots?</td></tr>
<tr><td>Communication skills tailored to addressing a range of vaccination concerns and motivators</td><td></td><td colspan="2">e.g. Did participants demonstrate these skills during the exercises?</td></tr>
<tr><td>Skills to effectively refute vaccine-related misconceptions in a sensitive way</td><td></td><td colspan="2">e.g. Did participants discuss these skills during the wrap up?</td></tr>
<tr><td>Awareness of tools and resources to help with vaccine communications</td><td></td><td colspan="2">e.g. Did participants note down the tools and resources?</td></tr>
<tr><td colspan="4">Delivery of module as intended (fidelity)</td></tr>
<tr><td colspan="4">1. Opening presentation/ demonstration of technique</td></tr>
<tr><td>- Attitude roots and empathetic refutation</td><td></td><td colspan="2">e.g. Did participants require clarification about the content presented?</td></tr>
<tr><td>- Using the jitsuvax.info website to look up attitude roots, affirmations, and tailored refutations</td><td></td><td colspan="2">e.g. Technical issues? Ease of navigation of website.</td></tr>
<tr><td>- Demonstrate interaction in a 5min video</td><td></td><td colspan="2">e.g. How did participants respond to the films?</td></tr>
<tr><td colspan="4">2. Role plays</td></tr>
<tr><td>-To what extent were participants able to use ERI in practice</td><td></td><td colspan="2">e.g. Were all participants able to use key steps</td></tr>
<tr><td>-Timings</td><td></td><td colspan="2">e.g. Did all participants have time to practice each role?</td></tr>
<tr><td colspan="4">3. Wrap up</td></tr>
<tr><td>Group conversation</td><td></td><td colspan="2">e.g. How well did the facilitator appear to elicit and respond to feedback</td></tr>
<tr><td>Reiterate key steps of ERI</td><td></td><td colspan="2">e.g. How was this provided</td></tr>
<tr><td>Where to find help and information, including other videos</td><td></td><td colspan="2">e.g. Did participants find the additional information sources useful?</td></tr>
<tr><td colspan="4">Adaptions and contextual observations that may affect implementation:</td></tr>
<tr><td colspan="4">Questions from participants during the session (note: include number of questions asked during session, as well as content)
(a) Number of questions: ____________
(b) Details of questions:</td></tr>
</table>
